# Cultural influences on palliative family caregiving: service recommendations specific to the Vietnamese in Canada

**DOI:** 10.1186/s13104-015-1252-3

**Published:** 2015-06-30

**Authors:** Allison M Williams, Rhonda Donovan, Kelli Stajduhar, Denise Spitzer

**Affiliations:** School of Geography and Earth Sciences, McMaster University, General Science Building, 209, 1280 Main Street West, Hamilton, ON L8S 4K1 Canada; Wilfrid Laurier University, Brantford Campus, RCE 216, 73 George Street, Brantford, ON N3T 2Y3 Canada; School of Nursing, University of Victoria, R Hut 129, Victoria, BC V8W 2Y2 Canada; Faculty of Social Science, Institute of Women’s Studies, University of Ottawa, Room 5034, 120 University Street, Ottawa, ON K1N 6N5 Canada

**Keywords:** Palliative caregiving, Culture, Program/policy implications, Vietnamese

## Abstract

**Background:**

Much of what is known about family caregiving at end-of-life in Canada has been studied within the context of various disease categories or across different care settings, rather than in relation to specific ethnic/cultural identities. Such homogeneity belies the impact of cultural and social factors on the experiences and outcomes of palliative and end-of-life (P/EOL) care. We know little about the end-of-life experiences of Vietnamese-Canadian families. Consequently, there is a lack of understanding around how to best meet the needs of Vietnamese care recipients, caregivers, and their families via the health service system, whose services of which we know they have limited access.

**Results:**

To determine a set of service recommendations for health care settings (including the home) specific to caring for Vietnamese (P/EOL) care recipients, caregivers and their families, a qualitative instrumental case-study design was employed. The perspectives of 18 adult Vietnamese family caregivers (FCGs) were obtained. In addition, seven semi-structured key informant interviews were implemented with a range of personnel from community service providers to front-line health care professionals. The ways in which caregiving was perceived and expressed were reflected in three thematic findings: (1) Natural: identity and care work; (2) Intentional: whole person care; and (3) Intensive: standards, struggle, and the context of care. Ten main recommendations have been vetted with service provider leaders and confirmed as being appropriate for uptake.

**Conclusions:**

The ten service recommendations for health care settings (including the home), if implemented, would contribute to improved P/EOL services for the Vietnamese population. Further research involves the evaluation of these policy and programs.

## Background

Much of what is known about family caregiving at end-of-life in Canada has been studied within the context of various disease categories or across different care settings, rather than in relation to specific ethnic/cultural identities. Such homogeneity belies the impact of cultural and social factors on the experiences and outcomes of palliative and end-of-life (P/EOL) care. We know little about the end-of-life experiences of Vietnamese-Canadian families. Consequently, there is a lack of understanding about how to best meet the needs of Vietnamese care recipients, caregivers, and their families via the health service system whose services of which we know they have limited access. Based on primary research conducted by the two leading authors, this article outlines a set of service recommendations for health care settings (including the home) specific to caring for Vietnamese (P/EOL) care recipients, caregivers and their families. These ten recommendations have been vetted with service provider leaders at a national conference workshop and confirmed as being appropriate for uptake.

Ethnic minority groups face problems accessing services for care and support, both in general and specific to P/EOL care. These problems include facing barriers to services due to: geographical [1]; linguistic, cultural or religious differences; and, different health beliefs [2–5]. In fact, research suggests that immigrant populations generally experience unmet health care needs, are unsure as to where to access services, and believe care will be inadequate [6]. The need to access culturally-appropriate health care may be heightened at end-of-life as people are more likely to draw upon various religious and cultural beliefs, practices and rituals in order to cope with the fear, stress, and grief associated with dying [2].

Differences exist in the experiences and outcomes for FCGs of diverse populations; however, the findings are limited. For example, much of what is known about diversity in caregiving has emanated from the United States, with comparisons between Euro-Canadian/American, African American and Hispanic populations caring for elderly dementia patients [7]. Comparatively fewer studies concerning Asian FCGs, particularly within the context of P/EOL care, exist in the literature, the majority of which derive from the UK. We have found no research that has focused on the experiences of Vietnamese FCGs specifically.

South Asians often prefer that care take place in the home by family members. For example Bowes and Wilkinson [8] found that South Asians held strong views against residential care, where it was seen as being both ideologically unacceptable and culturally-inappropriate. In addition, the context of family caregiving is often influenced by traditions and values specific to particular ethno-cultural identities [9, 10]. This includes values and beliefs which define perceptions and responsibility for care, how responsibilities are allocated, differences in how outcomes are reported, and differences in the use of formal services and informal support networks [7, 8, 11–13]. For example, perceptions around duties and obligations to care are often highly culturally-specific. Chinese and South Asian FCGs feel tremendous pressure to provide care and the caregiving role is seen as central to individual, gendered and group identity [12–14]. For example, in Chinese and South Asian cultures, the eldest son typically takes responsibility for care and decision-making, although the tasks of caregiving are usually performed by women [15].

Outcomes in caregiving vary between ethno-cultural groups [7]. For example, evidence suggests that higher levels of caregiver burden and depression are more common among Korean and Euro-Canadian/American caregivers than African-American caregivers. South Asian FCGs face tremendous pressure to provide care, and it is suggested that stress and strain are experienced in the same ways as in the general population [9]. The context of caregiving, which is typically mediated by normative behaviour and/or strong traditions for elder care, both buffers and engenders caregiver burden. Filial responsibility may buffer distress because caregiving work is not seen as a burden in this context; however, given the demands of care, FCGs’ inability to meet expectations and fulfill expected social roles may be a significant source of stress and guilt [13]. The use of formal services and informal networks of support tend to be culturally-specific, whereby those with stronger traditions towards family care, such as the case with South Asian and Chinese FCGS, often avoid external help [12, 13]. This may be due to fear of stigmatism or criticism for being unable to cope with familial responsibilities. It has been shown that political, social, cultural and economic processes intersect with ‘everyday and hidden spaces’ to both inform and circumscribe health practices and care work of migrant women [16, 17]. As such, within the context of their social and economic positions and with few networks of informal support, ethnic-minority FCGs may in fact find it necessary to seek formal help in cases of terminal-illness.

In addition to concerns for FCGs during the bereavement period, cultural norms may influence customs and rituals at the time of death; this has been so indicated for South Asian populations in particular, where care for the deceased body as well as rituals for burial and mourning prevail, as derived from religious beliefs and traditions [18–20]. To date, no studies directly exploring these issues in relation to the Asian experience of caregiving more generally, or the Vietnamese specifically, have been identified.

## Methods

As discussed at length by Donovan and Williams [21], the research informing the set of service recommendations for health care settings (including the home) specific to caring for Vietnamese (P/EOL) care recipients, caregivers and their families, employed a qualitative instrumental case-study design, where respondents were interviewed 1–5 times longitudinally. Face-to-face interviews captured data about caregiver supports, the caregiving experience, as well as cultural norms specific to caregiving, and ranged from 1 to 3 h in length. Cultural brokers/language interpreters were used to help ensure that the research was conducted in a culturally-appropriate manner. Using purpose snowball sampling, the sample was recruited through a wide range of community groups and organizations throughout southern Ontario, Canada (where the study took place). The sample included 7 bereaved and 11 active caregivers, all of whom were women and who had/were providing home-based care. Most caregivers spoke English; for those who didn’t, the interview was carried out primarily with the assistance of the cultural broker/language interpreter. Ethical approval for this study was obtained from the McMaster University Research Ethics Board (Protocol: 2009 078). A total of 18 FCGs providing care in the home were recruited to the study. Written informed consent was obtained for participation in this study. In addition, several key informant interviews (n = 7) estimating 1 h in length were conducted by the researchers (without the assistance of the cultural brokers/language interpreters); key-informants included community service providers and front-line health care professionals. Key-informants were only interviewed once; they were asked about the landscape of social/health service provision and cultural norms around caregiving for the Vietnamese. As described in detail by Donovan and Williams [21], Saldana’s [22] values and emotions coding was used to code the interview data.

## Results and discussion

Caregiving experiences were discussed within the context of a traditional cultural framework, influencing their motivations and approaches to caregiving, as well as their propensities towards the use of services and supports. As discussed at length by Donovan and Williams [21], the ways in which caregiving was perceived and expressed were reflected in three thematic findings: (1) Natural: identity and care work; (2) Intentional: whole person care; and (3) Intensive: standards, struggle, and the context of care [22]. The results illustrate how the caregiving experience, characterized as intensive given the function of cultural traditions, little social support, and the limited receipt of formal services, can negatively impact on the mental, physical and financial health of FCGs, and particularly women. Recognizing the limitations of a small sample size, no considerable differences were found within the research sample. It is important to note that although the research data informing the 10 recommendations below are from home-care specific data collected in southern Ontario, Canada, the recommendations have applicability across all health care settings and geographies. The ten recommendations have been vetted with palliative service provider leaders at a national workshop and confirmed as being appropriate for uptake.

1. Recognize that the decision to take on the informal caregiving role needs to be carefully negotiated given the culturally-informed expectation for women, in particular, to take on this role AND for the culturally-informed expectation that the home setting to be the ONLY option for care;
2. Recognize that formal health care providers are often the ONLY conduit for information about the service landscape and thereby KEY to creating awareness of the range of services available;
3. Recognize the importance of trust-building over TIME and PLACE; this requires CONTINUITY in services/human resources, particularly when hands-on personal care is required. This will ensure the development of relationships with providers, including meaningful methods of communication;
4. Recognize the need for the provision of POSITIVE affirmation with respect to the quality of care being provided by those informal caregivers who elect to do so, as they take PRIDE in their role and the quality/quantity of care provided;
5. Given the silent suffering and few social supports experienced by caregivers, recognize the need for ‘SELF-CARE INTERVENTIONS’, such as exercise, meditation, me-time, hobbies, social opportunities/activities, required/available respite periods, etc. AS WELL as culturally-specific Social Workers/Community Workers available to talk with/to on a regular basis;
6. Recognize families generally have FEW economic resources, impacting out-of-pocket expenses; provide a sliding scale option OR offer available supplements for those with few financial resources;
7. Encourage the use of whole-person care (PALLIATIVE) versus GENERAL care services given the cultural expectation to meet whole-person care (emotional, physical, spiritual, psycho-social). Related to this, recognize and accept the strong likelihood of patients using both bio-medical AND alternative/traditional care types;
8. Recognize that FOOD has major symbolic and practical relevance, suggesting its integration into the care plan;
9. Recognize the use of LANGUAGE/CULTURAL-specific print/audio-visual materials wherever possible, given the importance of sharing information about services and caregiving knowledge;
10. Recognize the use of CULTURAL BROKERS in all medical/health encounters, or at least the availability of interpreters in medical/health care encounters and via tele-health services.

## Conclusions

The ten service recommendations for health care settings, if implemented, would contribute to improved P/EOL services for the Vietnamese population. Further research involves the evaluation of these policy and programs.

## Abbreviations

P/EOL: palliative and end-of-life
FCGs: family caregivers
